# Central place foragers select ocean surface convergent features despite differing foraging strategies

**DOI:** 10.1038/s41598-018-35901-7

**Published:** 2019-01-17

**Authors:** Matthew J. Oliver, Josh T. Kohut, Kim Bernard, William Fraser, Peter Winsor, Hank Statscewich, Erick Fredj, Megan Cimino, Donna Patterson-Fraser, Filipa Carvalho

**Affiliations:** 1 University of Delaware, College of Earth, Ocean and Environment, 700 Pilottown Road, Lewes, DE 19958 USA; 2 Rutgers, The State University of New Jersey, Department of Marine and Coastal Sciences, 71 Dudley Road, New Brunswick, NJ 08901 USA; 3 Oregon State University, College of Earth, Ocean, and Atmospheric Sciences, 104 CEOAS Admin Bldg, Corvallis, OR 97330 USA; 4 Polar Oceans Research Group, P.O. Box 368, Sheridan, MT 59749 USA; 5 University of Alaska, Fairbanks, College of Fisheries and Ocean Sciences, 905 Koyukuk Dr. Suite 245 O’Neill Bldg., Fairbanks, AK 99775–7220 USA; 6 The Jerusalem College of Technology, Computer Science Department, 21 Havaad Haleumi St., P.O. Box 16031, Jerusalem, 91160 Israel; 7 Scripps Institution of Oceanography, University of California, San Diego, Coastal Observing R&D Center, 9500 Gilman Drive #0214, La Jolla, CA 92093 USA; 8 National Oceanography Centre, European Way, Southampton, SO14 3ZH United Kingdom

## Abstract

Discovering the predictors of foraging locations can be challenging, and is often the critical missing piece for interpreting the ecological significance of observed movement patterns of predators. This is especially true in dynamic coastal marine systems, where planktonic food resources are diffuse and must be either physically or biologically concentrated to support upper trophic levels. In the Western Antarctic Peninsula, recent climate change has created new foraging sympatry between Adélie (*Pygoscelis adeliae*) and gentoo (*P. papua*) penguins in a known biological hotspot near Palmer Deep canyon. We used this recent sympatry as an opportunity to investigate how dynamic local oceanographic features affect aspects of the foraging ecology of these two species. Simulated particle trajectories from measured surface currents were used to investigate the co-occurrence of convergent ocean features and penguin foraging locations. Adélie penguin diving activity was restricted to the upper mixed layer, while gentoo penguins often foraged much deeper than the mixed layer, suggesting that Adélie penguins may be more responsive to dynamic surface convergent features compared to gentoo penguins. We found that, despite large differences in diving and foraging behavior, both shallow-diving Adélie and deeper-diving gentoo penguins strongly selected for surface convergent features. Furthermore, there was no difference in selectivity for shallow- versus deep-diving gentoo penguins. Our results suggest that these two mesopredators are selecting surface convergent features, however, how these surface signals are related to subsurface prey fields is unknown.

## Introduction

Optimal foraging theory suggests central place foragers consider external cues like food quality, distance to food patch, and revisit times to food patches to maximize fitness^1^. The end result of the feedback between prey patch characteristics and the desire and ability of the predator to find food is manifested as random walks^2^, Lévy walks^3^, or other diffusive^4^ or multi-modal movements. Interpreting the ecological significance of these movement modes necessitates an understanding of the dynamic nature of the available environmental cues, the relative response of predators and prey to these cues, and how organisms remember these cues^5^. For example, many organisms appear to exhibit Lévy walks, which are documented to optimize foraging success of random searchers^6^. However, the selective interactions that lead to the emergence of these patterns are often unknown, hence in the absence of an understanding of selective cues between the environment and the focal individual, alternative movement modes may equally explain observed movement patterns^7,8^. Therefore, it is not enough to only establish a movement mode to understand the ecological significance of foraging behaviors or how these behaviors might change in dynamic environmental conditions. Discovering the environmental predictors of foraging locations is equally important, yet can be challenging and is often the critical missing piece for interpreting the ecological significance of observed movement patterns of predators. It is difficult to map environmental cues at the appropriate scale to determine if they are being selected^9,10^, especially in a fluid marine environment. In the coastal Western Antarctic Peninsula (WAP), the food web is short and characterized by intense phytoplankton blooms that are grazed by Antarctic krill (*Euphausia superba*, referred to hereafter as “krill”), a primary prey source for penguins and other predators. Although krill aggregations occur throughout the WAP^11^, their distribution is extremely patchy even on scales less than 1 km^12,13^. Krill have intermediate Reynolds numbers (~10^2^–10^3^), compared to drifting phytoplankton (~10^−2^) and swimming penguins (~10^6^), which means they can make directed movements, even though they are also heavily influenced by local circulation.

Lagrangian convergent features are representations of time-dependent concentrating ocean dynamics at scales relevant to marine predator foraging ecology. Broadly, they are regions that concentrate neutrally buoyant particles and are often associated with filaments and mesoscale features, such as eddies, jets and fronts. Convergent features, identified by time varying concentrations of buoyant particle densities, may be proxies for the mechanisms by which sparse food resources move through marine trophic levels by collapsing the essential components of the food web in time and space. Realistic particle simulations show that convergent features have much higher concentrations of zooplankton^14^. Seabirds have also been associated with convergent features such as mesoscale eddies^15^, and their flight paths are coherent with dynamic convergent features^16^. Macaroni penguins have been shown to associate with convergent features at relatively large scales (10–100 km), presumably because they concentrate prey resources^17^. These studies have focused on relatively large-scale associations using infrequent satellite composites of ocean features. However, convergent features are dynamic in space and time, and therefore should optimally be examined at space and time scales relevant to predator foraging behaviors.

**Figure 1:**
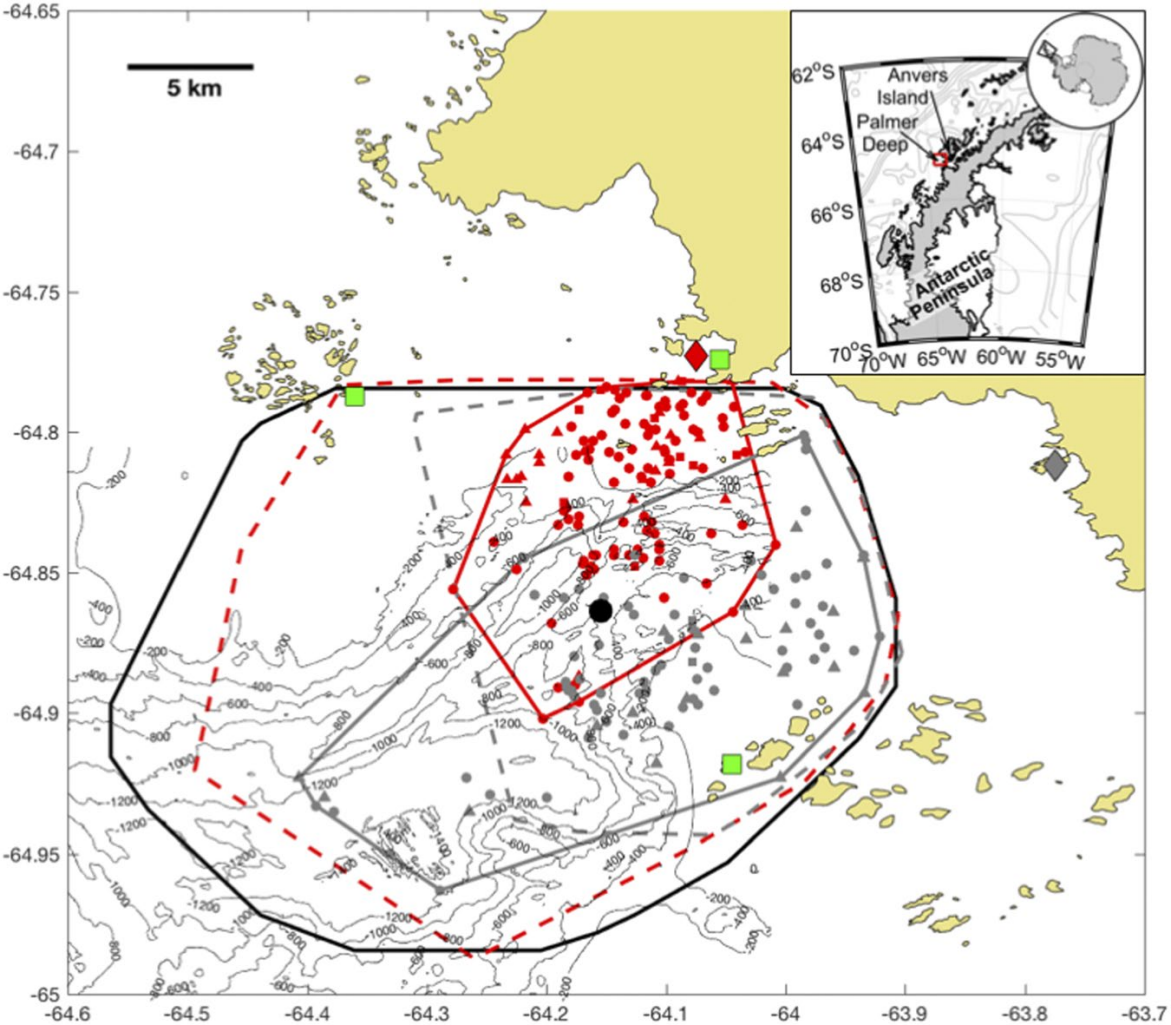
Map of the study site over Palmer Deep. Locations of tagged Adélie (red) and Gentoo Penguin (grey) forage dives (circles), search dives (squares) and transits (triangles) for 2015 study season are shown. The convex spatial hulls are shown for the tagged (thin red) and simulated (red dashed) Adélie and the tagged (thin grey) and simulated (grey dashed) Gentoo Penguins for the 2015 study season. Also shown is the Adélie colony at Torgersen (red diamond) and the gentoo colony at Biscoe Point (grey diamond); the HFR sites (green squares); the HFR data footprint (thin black line); and the glider time series (black circle). The maps were generated by the authors J.K. and H.S. using Matlab version R2016b (www.mathworks.com).

At our study site, Adélie penguin (*Pygoscelis adeliae*) populations have decreased rapidly over the last three decades, while numbers of gentoo penguins (*P. papua*) have increased^18^. These population changes have resulted in relatively recent sympatry between these two congeneric central place foragers in our study site, where they also exhibit partially overlapping foraging ranges over the Palmer Deep canyon (Fig. 1). In this study, we determine if these two species select convergent features in a similar way to guide their foraging behavior. To do this, we used an integrated ocean observatory (Fig. 1) to estimate the relationship between surface convergent features and the foraging behavior of these two mesopredators.

## Results

### Penguin Locations Relative to Ocean Features

**Figure 2:**
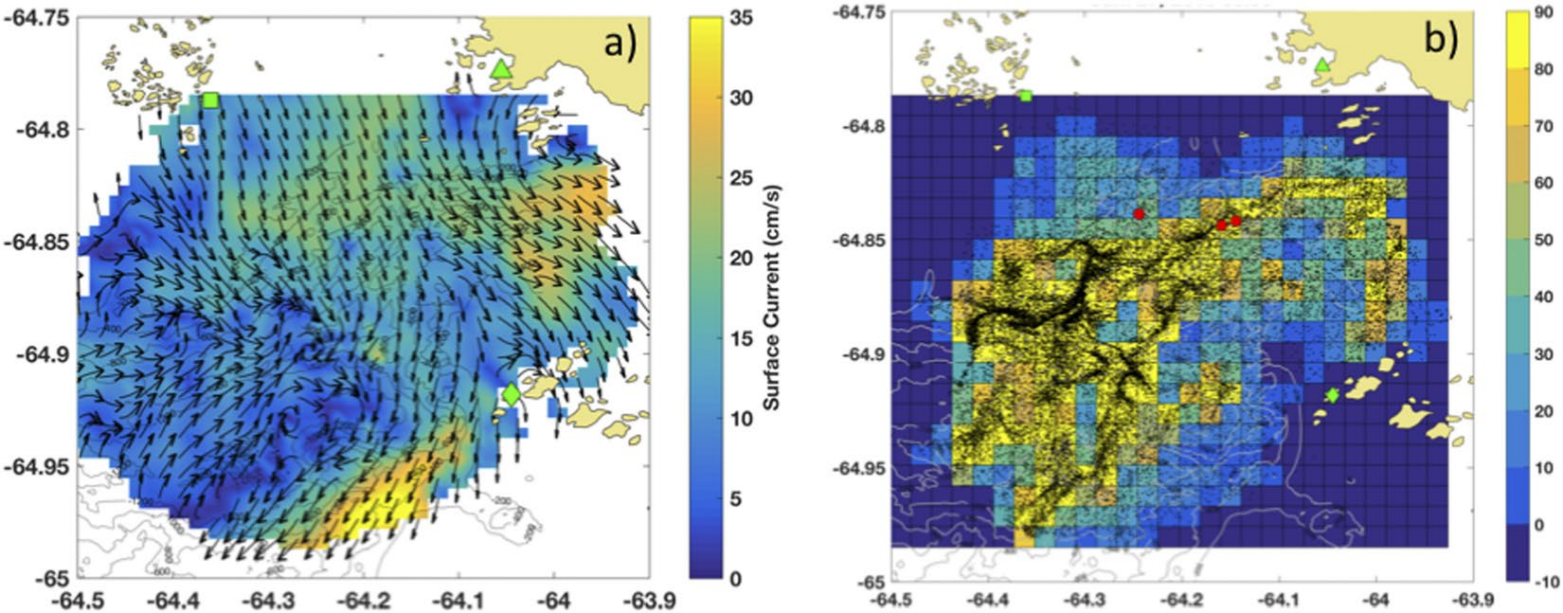
Convergent features near penguin foraging. (**a**) Hourly surface current map, January 27, 08:00 GMT 2015. The HFR sites located at Palmer Station (green triangle) and the Wauwermans (green diamond) and Joubin (green square) island groups are also shown. (**b**) Map showing the distribution of particles on January 27, 08:00 GMT (black dots) overlaid on the particle density metric (number of particles within each 1 × 1 km cell minus the median across all cells). The location of penguins is also shown (red circles). The maps were generated by the corresponding author J.K. using Matlab version R2016b (www.mathworks.com).

During the austral summer of 2014–2015, we mapped penguin foraging patterns relative to sea surface currents derived each hour from a High Frequency Radar (HFR) network over Palmer Deep canyon (Fig. 2a)^19^. Simulated passive particles released in the hourly surface current maps were used to identify the location and intensity of convergent features that may locally concentrate prey biomass during penguin foraging days. Maps of Relative Particle Densities (RPD) derived from particles released each hour across the HFR footprint were used to estimate the location of convergent features each hour between January 1^st^ and March 1^st^ 2015, where higher RPD values were indicative of convergence (Fig. 2b).

**Figure 3:**
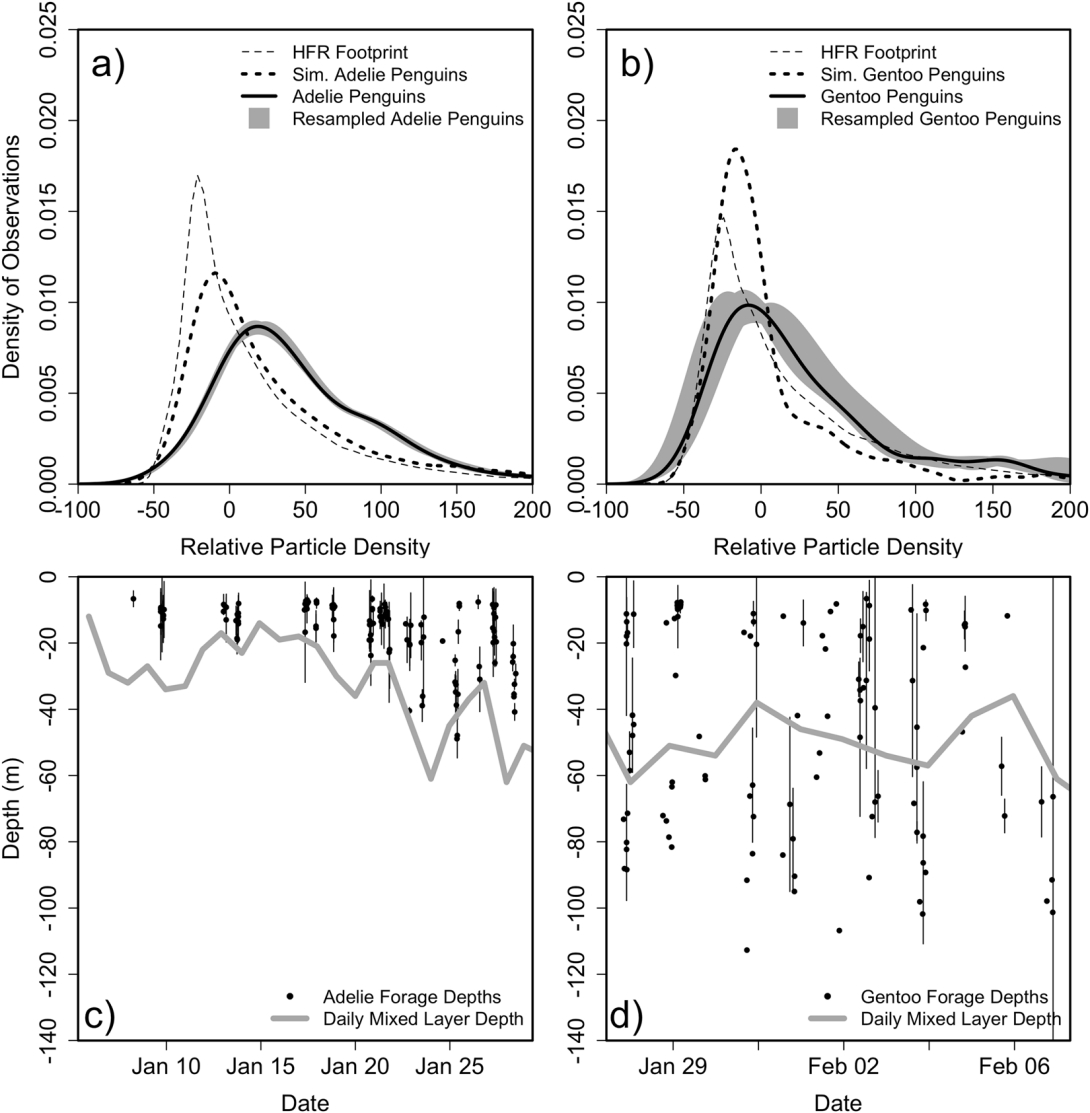
Penguin selectivity relative to convergent ocean features. Distribution of observed field PD values available to the penguins (grey dashed) and distribution of PD values at the tagged (solid black) and simulated (black dashed) penguin dive location for the (**a**) Adélie and (**b**) Gentoo Penguins. The range of solutions with of the resampled penguins by individual and by foraging trip is shown as a gray shade. Foraging dive depths (black dots) with one standard deviation and daily mixed layer depth determined from the station keeping glider (grey line) for the (**c**) Adélie and (**d**) gentoo penguins.

For 11 Adélie and 7 gentoo penguins, there was a total of 124 and 98 ARGOS class 1–3 locations representing 27 and 17 foraging trips, respectively. These locations were spatially matched to hourly RPD (Tables 1 and 2). ARGOS locations were classified as transiting, searching or foraging based on their dive profiles within 1, 2.5, 5, and 15 minutes of the ARGOS location (Tables 1 and 2). Adélie penguin dives associated with ARGOS locations had significantly higher RPD values compared to RPD values across the entire HFR field and significantly higher RPD values compared to the RPD values sampled by simulated Adélie penguins (p ≪ 0.001 and p ≪ 0.001, two-sample Kolmogorov-Smirnov tests respectively, Fig. 3a). Gentoo penguin locations had higher RPD values compared to RPD values across the entire HFR field but these were weakly significant (p = 0.03, two-sample Kolmogorov-Smirnov tests respectively, Fig. 3b). However, compared to RPD values sampled by simulated gentoo penguins, tagged gentoo penguins showed a significant selection for higher values as well (p ≪ 0.001, two-sample Kolmogorov-Smirnov tests, Fig. 3b).

**Table 1:** The p-value and number of observations for KS tests comparing the field RPD to Adélie and gentoo ARGOS locations classified into transiting, search diving and forage diving behavior.

Species	Time Window (minutes)	Transiting	Search Diving	Forage Diving
Adélie	1	**0.001 (N = 32)**	**≪0.001 (N = 18)**	**≪0.001 (N = 74)**
	2.5	**≪0.001 (N = 22)**	**0.004 (N = 8)**	**≪0.001 (N = 94)**
	5	**0.004 (N = 16)**	**0.004 (N = 7)**	**≪0.001 (N = 101)**
	15	0.184 (N = 2)	0.307 (N = 4)	**≪0.001 (N = 118)**
Gentoo	1	**0.009 (N = 49)**	0.760 (N = 7)	0.309 (N = 42)
	2.5	0.373 (N = 22)	0.460 (N = 4)	0.063 (N = 72)
	5	0.547 (N = 10)	0.836 (N = 2)	**0.007 (N = 86)**
	15	0.835 (N = 2)	N = 0	**0.009 (N = 94)**

The classifications were based on 1, 2.5, 5, and 15 minute windows before and after the ARGOS hit to classify the location. Bold text indicates that ARGOS locations had higher RPD than the field RPD.

**Table 2:** The p-value and number of observations for KS tests comparing the RPD of simulated Adélie or gentoo penguins to Adélie or gentoo ARGOS locations classified into transiting, search diving and forage diving behavior.

Species	Time Window (minutes)	Transiting	Search Diving	Forage Diving
Adélie	1	0.075 (N = 32)	**0.001 (N = 18)**	**≪0.001 (N = 74)**
	2.5	**0.028 (N = 22)**	**0.017 (N = 8)**	**≪0.001 (N = 94)**
	5	0.057 (N = 16)	**0.021 (N = 7)**	**≪0.001 (N = 101**)
	15	0.294 (N = 2)	0.543 (N = 4)	**≪0.001 (N = 118)**
Gentoo	1	**≪0.001 (N = 49)**	0.339 (N = 7)	**0.025 (N = 42)**
	2.5	0.120 (N = 22)	0.189 (N = 4)	**≪0.001 (N = 72)**
	5	0.466 (N = 10)	0.821 (N = 2)	**≪0.001 (N = 86)**
	15	0.874 (N = 2)	N = 0	**≪0.001 (N = 94)**

The classifications were based on 1, 2.5, 5, and 15 minute windows before and after the ARGOS hit to classify the location. Bold text indicates that ARGOS locations had higher RPD than the simulated penguins RPD.

Both simulated and tagged penguins covered different areas of the HFR field during our experiment, and therefore experienced different RPD. However, simulated penguins within the convex hull of their tagged counterparts still showed significantly lower RPD values compared to tagged Adélie and gentoo penguins (p = 0.02 and p = 0.0005, respectively). To test the sensitivity of these results to the effects of individual trips or individual penguins, we resampled these ARGOS locations, systematically leaving out one foraging trip or one penguin (represented by the grey envelope in Fig. 3a,b). In all resampling cases, the Adélie and gentoo ARGOS RPD values were significantly higher than both the field and simulated penguin RPD values (p ≪ 0.001, two-sample Kolmogorov-Smirnov tests). As a result, it is likely that both Adélie and gentoo penguins were systematically selecting for higher RPD.

We partitioned the ARGOS locations by behavior classification within the above mentioned time intervals (Tables 1 and 2). For Adélie penguins, ARGOS locations classified as foraging and search diving behavior occurred in significantly higher RPD compared to both the background RPD and the simulated penguin RPD. The exception was for the search diving behavior classified with a 15 minute time window, likely due to a low sample size (n = 4). Adélie penguin ARGOS locations classified as transiting had higher RPD compared to the background RPD, except when using a 15 minute time window due to low sample size (n = 2). Furthermore, only RPD for transiting Adélie penguins classified with the 2.5 minute window were significantly higher than the simulated penguins, although it should be noted that for the shorter time window of 1 minute the p-value was 0.075. For the tagged gentoo penguins, only ARGOS locations classified as forage diving had significantly higher RPD compared to the simulated penguin RPD for all time classifications. Search diving behavior had very few samples. The largest sample size for ARGOS locations classified as transiting with a 1 minute time window had significantly higher RPD compared to both background and simulated penguin RPD, while the longer time windows with fewer samples did not have significantly higher RPDs compared to both the background and simulated RPD. Overall, only ARGOS locations classified as forage diving had significantly higher RPD compared to simulated penguin RPD across both species and all time windows (Table 2).

Adélie and gentoo penguins displayed markedly different diving behaviors relative to oceanographic features. Adélie penguins foraged in waters above the mixed layer at depths between 5 and 50 m, where the surface layer is sensitive to atmospheric forcing, while gentoo penguins foraged both above and below the mixed layer, at depths between 5 and 100 m (Fig. 3c,d). The mixed layer depth (MLD), estimated from the maximum buoyancy frequency^20^, was ~30 m during the time period that Adélie penguins were tagged and deepened to ~50 m during the period gentoos were tagged, which accords with natural seasonal changes^21^. We partitioned the gentoo data by dive depth relative to MLD. Gentoo penguins diving shallower than the MLD selected for higher RPD values than the simulated gentoo penguins (p = 0.028). Gentoo penguins diving deeper than the MLD also selected for higher RPD values compared to the simulated gentoo penguins (p ≪ 0.001). The distributions of RPD targeted by the shallow and deep diving penguins were not significantly different (p = 0.1), indicating that gentoo selectivity of surface RPD was not dependent on penguin foraging depth.

### Relative Particle Density Values in Diurnal and Semi-Diurnal Tidal Regimes

**Figure 4:**
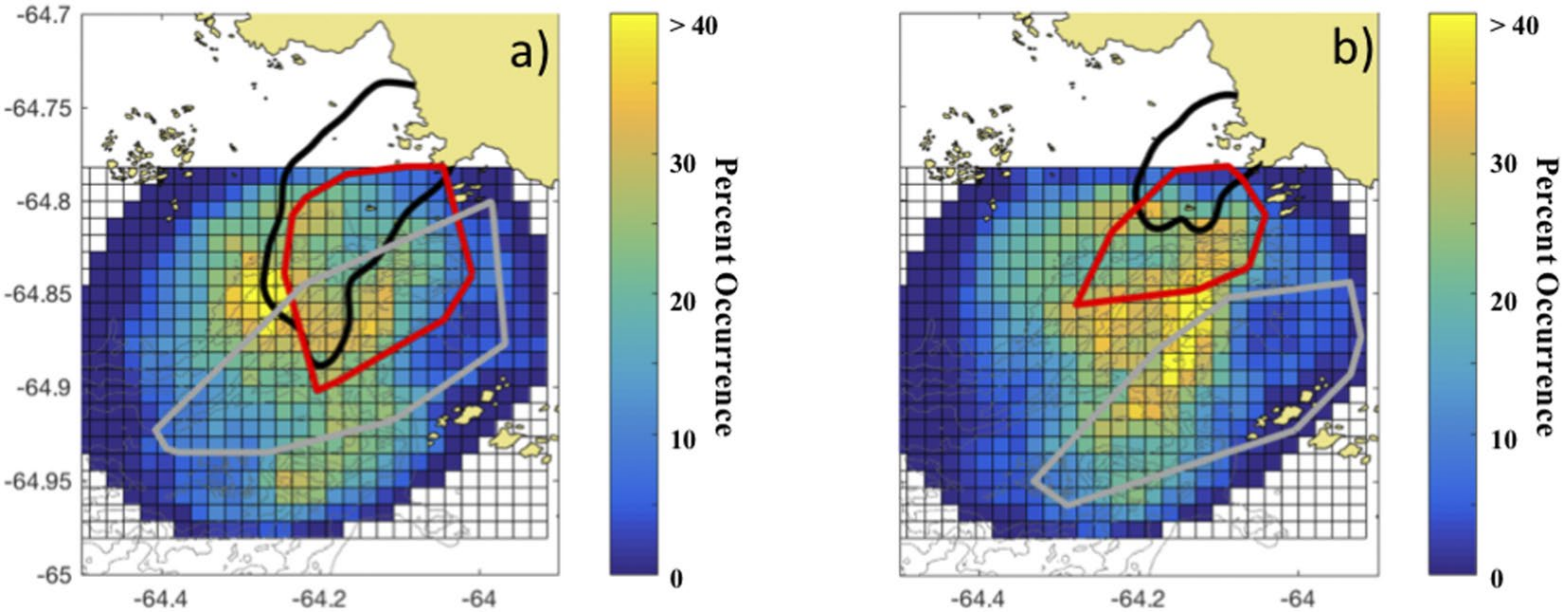
Maps of the percent of occurrence of the convergent features based on RPD (number of particles minus median > 100) observed during (**a**) semi-diurnal and (**b**) diurnal days. The spatial density kernels (black contours) based on 10 years of tagged Adélie data are shown in panel a) for the semi-diurnal days and in panel b) for diurnal days^25^. The convex hulls for the 2015 Adélie (red) and gentoo (grey) ARGOS locations are also shown. In 2015, Adélie penguins also foraged closer to their home colony during diurnal, compared to semi-diurnal tides. The maps were generated by the corresponding author J.K. using Matlab version R2016b (www.mathworks.com).

Prior work has shown that local tides are coherent with Adélie penguin ARGOS locations over Palmer Deep^22^. The dominant tidal constituent near Palmer Station is the diurnal K_1_ followed closely by another diurnal constituent, O_1_. The two prominent semi-diurnal constituents, K_2_ and M_2_, are smaller in magnitude^23^. The interaction of these constituents leads to a unique mixed tide that is slightly diurnal-dominated based on tide gauge data collected at Palmer Station^23^. The result is a tidal forcing and response that transitions from a diurnal to semi-diurnal regime approximately every two weeks. A long-term (2002–2011) record of Adélie penguin foraging distances showed that Adélies foraged at greater distances from shore during semi-diurnal tides, compared to diurnal tides (Fig. 4)^22^. In 2015, Adélie ARGOS locations expanded to the south and east during semi-diurnal tides, while gentoo ARGOS locations translated to the north and west during semi-diurnal tides. During the austral summer of 2014–2015, we mapped the location of strong convergent features based on the hourly RPD maps partitioned by days with semi-diurnal and diurnal tides. The convergent features were defined as grid cells with particle counts, normalized by subtracting the spatial median, greater than 100. The location frequency of these strongest fronts associated with the semi-diurnal and diurnal tidal regimes are shown in Fig. 4. During the semi-diurnal tidal regime, the highest occurrence of convergent features was located offshore over the central canyon (Fig. 4a), consistent with the offshore historic Adélie penguin foraging locations observed during semi-diurnal tides. During the diurnal tidal regime, the location of the highest percentage of convergent features moved south and inshore (Fig. 4b), closer to the penguin colonies. We suggest that the predictability of the locations of convergent features associated with the changing tidal regimes combined with their selection for these convergent features provides a mechanistic explanation for the variability in historic foraging locations observed in Adélie penguins^22^.

## Discussion

The outer and mid WAP continental shelf is characterized by irregular, episodic intrusions of Upper Circumpolar Deep Water (UCDW)^24,25^ that drive intense phytoplankton blooms that may be advected into coastal regions^26,27^. These blooms are fed on by krill, which show a high degree of interannual variability in their abundance^28,29^, both along the shelf and into coastal regions^30–32^. Palmer Deep canyon supports enhanced phytoplankton production^33^ and is considered a biological hotspot that is home to Adélie, gentoo and chinstrap penguins^34^. This region is also a common feeding ground for humpback whales (*Megaptera novaeangliae*)^35^, indicating that it is a place where upper trophic levels are persistently linked to primary producers through krill and other zooplankton. What is not known are the specific physical mechanisms that concentrate the various levels of the food web at the scales of the individual predators.

Within the Palmer Deep hotspot, a historical analysis (10 years) of Adélie penguin foraging locations demonstrated a correlation to local tidal regimes, indicating that individual penguins may track tidally-driven features, such as convergent zones, associated with the diurnal and semi-diurnal tides^19,22^. Here we show that as the tidal regime shifts from diurnal to semi-diurnal, the spatial patterns in the occurrence of strong convergent features is matched by similar shifts in penguin foraging locations. While penguin foraging locations and the occurrence of convergent features covaried with tidal regime (Fig. 4), we show that both Adélie and gentoo penguins were specifically selecting for stronger surface convergent zones than were available based on simulations, suggesting the importance of these features at the scale of the individual. Despite the different typical dive depths exhibited by these two species, both the shallow-diving Adélie and deeper-diving gentoo penguins selected for surface convergent features (Fig. 3).

Penguins in the Palmer Deep canyon region travel relatively short distances (~8–25 km) during foraging trips compared to penguins breeding in many other locations, where foraging distances may reach up to 100 km^36^. These short foraging distances are also combined with the persistence of foraging locations across both tidal regimes, suggesting that these penguins may not need to use environmental cues to initiate foraging behavior; that is, they are simply returning to the same general location to find prey because the prey field spans the entire area. However, we argue that this is not always the case. Although the prey-scape was not spatially resolved in this study, previous vessel and AUV acoustic surveys in the region report typical krill patch length scales on the order of 40 m^12,37^. These patches are dispersed hundreds of meters apart across the +20 km foraging range of the local Adélie and gentoo colonies^12^. This spacing by itself might suggest that both Adélie and gentoo penguins could return to the same foraging ground and be successful, independent of foraging cues. However, measurements of the residence time of this region indicate that the surface layer is replaced on average every two days, and can be as short as 18 hours^38^. This leads to a very patchy and rapidly evolving prey environment within the penguin foraging range that is simultaneously targeted by other species including whales and seals. Therefore, the rapid replacement of the surface layer may necessitate individual foraging responses triggered by oceanographic conditions at the scale of the individual, along with memory of recent successful foraging trips, or social cues from other foragers^5^.

At Palmer Station, Adélie penguins are often relatively shallow divers (<50 m)^39^ compared to gentoo penguins that often dive deeper (<100 m)^37,40^. Gentoo penguins are the larger of the two species and consequently have a greater scope for maximum diving depth, even though Adélie penguins are capable of dives to similar depths^41^. During our study, Adélie penguins not only selected for stronger surface convergent features compared to their availability, but their foraging was also limited to the surface mixed layer. In contrast, gentoo penguins foraged both above and below the surface mixed layer, with some dives as deep as 150 m, yet also selected for stronger surface convergent features. We suggest that despite the variable foraging dive depths relative to the mixed layer, both species use surface layer convergent features as foraging cues.

ARGOS locations associated with foraging behavior for both species had consistently higher RPD compared to simulated penguins and background RPD suggesting that convergent features may cue foraging behavior. What is less clear is the impact of higher RPD for transiting Adélie and gentoo penguins. Gentoo penguins showed little selectivity for increased RPD during transiting behavior, with only one exception, suggesting that perhaps gentoo penguins are using past foraging experiences to get to a general foraging region before selecting for higher RPD values at finer spatial scales. Transiting Adélie penguins, however, showed some selectivity for higher RPD compared to background concentrations. As the time window we used to behaviorally classify ARGOS locations widened (Table 1), all but two ARGOS locations were considered to be associated with foraging behavior, suggesting foraging behavior could be interspersed throughout a foraging trip. It has been shown previously that ARGOS locations without diving behavior were strongly coherent with ARGOS locations with diving behavior in this location for Adélie penguins^22^.

The link between the occurrence of strong convergent features and the foraging behavior of satellite-tagged penguins raises important questions about the coupling mechanisms operating throughout the entire food web. These convergent features may coincidently concentrate or attract krill in the surface layer, and trigger penguin foraging behavior, irrespective of whether penguins are shallow- or deep-diving. This would be consistent with a penguin that repeatedly dives in the same location once a prey patch is found^5^. Alternatively, there may be different physical mechanisms that concentrate prey above and below the mixed layer. For example, barotropic tides influence the entire water column, while the seasonal surface mixed layer circulation is likely driven by local winds^38^ but retains a tidal signal when integrated over the foraging season. Below the mixed layer, circulation is likely driven by the bathymetric steering of density currents along isobaths (i.e. along f/H contours), suggesting the influence of the Palmer Deep canyon. Critically, these depth-dependent features could be co-located with deeper gentoo prey aggregations, thus explaining why deep-diving gentoos appear to be selecting for higher surface convergence, even though they are feeding well below the surface mixed layer. Another speculative possibility that could explain why deep diving penguins selected for higher surface RPD, is that they may select convergent features independently of visual prey detection. Dimethylsulphoniopropionate is released from grazed phytoplankton, which is volatilized in the ocean surface as dimethyl sulphide (DMS)^42^, and is not necessarily correlated to surface phytoplankton concentrations^43^. Krill-consuming chinstrap penguins (*P. antarctica*), for example, have been shown to be attracted to DMS^44^, and African penguins (*Spheniscus demersus*) have been shown to use DMS as a foraging cue^45^. If surface RPD values are a proxy for higher DMS, this may explain why deep diving gentoo penguins select for higher RPD values.

Given our results, we believe that physical factors like surface convergent features are an important mechanism that influences penguin foraging locations, and are therefore a critical feature influencing the maintenance of the Palmer Deep biological hotspot for penguins. Convergent fronts likely concentrate krill, the primary prey of penguins^46^ in this region. This example of tight coupling from the hydrography through the lower trophic levels to foraging penguins shows the important role that these physical features may have on the coastal Antarctic food web. If these features are commonly targeted by predators, they may represent a key physical mechanism that is critical for the persistence of the Palmer Deep biological hotspot over the last 1000 years^34^, despite known climate and environmental variability. However, because this study did not simultaneously resolve the distribution of krill and all of their major predators, there is still much work to be done to understand how important prey convergence is relative to other factors affecting prey distributions. Even though both penguin species selected, on average, for higher convergence zones, both also utilized a wide range of particle densities. This suggests to us that there are other important factors, in addition to surface convergence, affecting foraging behavior. One possibility is the top-down impact of other krill predators. For example, Adélie penguins at Cape Crozier in the Ross Sea increased their foraging duration and dove deeper as krill were removed by predation near the colony^47^, suggesting a significant top-down control on penguin foraging location. In our study, we also observed a deepening of forage depths by Adélie penguins (Fig. 3c). Because this study did not resolve the distribution of krill, or account for other krill predators like whales, it is difficult for us to tell if prey depletion was an important factor in this study. One important difference between the colonies at Cape Crozier and those at Palmer Deep is that of colony size; the colonies in the Ross Sea that showed the prey depletion effect are two orders of magnitude larger than those near Palmer Deep. Because of this, we speculate that top-down effects like prey depletion play a relatively smaller role in determining foraging location near Palmer Deep. However, the relative importance of bottom up physical concentration factors and top down biological factors on penguin foraging remains an open and important question for understanding how these ecosystems may change in the future.

## Materials and Methods

### High Frequency Radar (HFR)

HFR systems, typically deployed along the coast use Bragg peaks within a transmitted signal (3~30 MHz) scattered off the ocean surface to calculate radial components of the surface velocity at a given location^48^. Individual sites, composed of electronics, cables and a transmit and receive antenna, generate maps of surface component vectors directed toward the antenna with range resolution of 500 m horizontally and 5 degrees in azimuth. To provide sufficient coverage over the penguin foraging grounds associated with Palmer Deep, a three-site HFR network was deployed in November 2014 (Fig. 2). The first site was deployed at and powered by Palmer Station. The other two sites, deployed at the Joubin and Wauwermans Islands, relied on remote power systems that were constructed on site, lightered to shore via zodiac with ship support. Remote Power Modules (RPMs) generated the required power for the HFRs through a combination of small-scale micro wind turbines and a photovoltaic array with a 96-hour battery backup^19^. The RPMs consisted of a single watertight enclosure, used to house power distribution equipment, the HFR, and the communication gear. Built-in redundancies within the RPMs, including wind charging/resistive loads, solar energy, and independent battery banks ensured that, should any one component fail, the unit would be able to adjust autonomously. Each site also collected 15-minute meteorological measurements of air temperature, wind, relative humidity, and solar radiation. Communication between the two remote sites and Palmer Station was with line of sight radio modems (Freewave), which enabled remote site diagnostics and maintenance and provided a real-time data link.

The three-site network collected hourly measurements of ocean surface current component vectors throughout the penguin foraging season (November 2014 through March 2015). Every hour, the radial components from each site were combined into two-dimensional vector maps using the optimal interpolation algorithm of^49^. Throughout the time of active penguin foraging, a roughly 1,500 km2 area of ocean over Palmer Deep was covered greater than 80% of the time with hourly maps of surface ocean circulation. The evolution of these current fields was used to identify convergent features, including fronts and eddies, relative to known penguin foraging.

### Surface Convergent Features

Various metrics have been used to map ocean convergent features. Maps of Lagrangian Coherent Structures (LCS), specifically Finite-Time Lyapunov Exponent (FTLE) and Finite-Space Lyapunov Exponent (FSLE) have seen greater application to marine ecological studies^14,16,50,51^. While these metrics often delineate boundaries in a fluid that distinguish regions with differing dynamics^52^ they are based on an assumption that the input velocity fields are horizontally non-divergent (i.e. zero vertical velocity). The highly resolved current maps provided by the HFR network deployed over Palmer Deep display complex currents that do not meet this important criterium for both FTLE and FSLE. Consequently, we define a more appropriate metric to map the convergent features within our study site consistent with the dynamics captured by the HFR surface current maps.

To objectively map the time and location of convergent ocean features in the mapped surface current time series, we used a metric derived from simulated particle releases in the HFR surface current fields. Our relative particle density (RPD) metric is calculated based on the movement of simulated particles released in the HFR footprint and tracked over time. Each hour, we released simulated particles over a 200 × 200 m grid over the HFR footprint. The Lagrangian particles were advected in the HFR velocity field with a 4^th^-order Runge-Kutta integration scheme for a period of 48 hours. In our application, we compute hourly maps of RPD from *t*_1_ = December 31, 2014, to *t*_*N*_ = February 19, 2015 (spanning the date range that penguins were tagged and actively foraging).

The hourly RPD was determined by the number of particles within 1 × 1 km boxes within the overlapping HFR coverage and penguin foraging grounds (Fig. 2b). To minimize the effect of the grid on the particle densities, only particles in the field for at least 6 hours were included in the count. To correct for time varying residence time of particles throughout the study period^38^, each count was normalized by subtracting the median count across all 1 km boxes within the field for each time step (Fig. 2b), termed RPD for the purposes of this analysis.

### Slocum Glider

Gliders are buoyancy driven vehicles that dive and climb at a nominal 26° angle and travel in a vertical “sawtooth” pattern between predetermined surface events 49. Glider-based sampling provided a continuous presence, through all weather conditions, over the spatial domain identified by the HFR network. Simultaneous measurements of physical and biological variables from the gliders sampled the spatial and temporal variability over Palmer Deep. A glider was programmed to complete a mission as a virtual mooring between January 5, 2015 to February 26, 2015 (Fig. 1), diving between the surface and 100 m. The glider was equipped with a sensor suite to characterize the ecosystem’s physical structure (Seabird C, T, D). This glider provided the time series of mixed layer depth throughout the penguin foraging time period used in this analysis^20^.

### Penguin Tagging and Dive Analysis

From January 5 to 28, 2015, we deployed ARGOS satellite transmitters on 12 Adélie penguins (8 female, 4 male) that nested on Torgersen Island (64°46′S, 64°5′W), and from January 27 to February 7, 2015, we deployed satellite transmitters on 7 gentoo penguins (2 female, 5 male) at Biscoe Point (64°49′S, 63°46′W). All protocols were carried out in accordance with the approved guidelines of the Columbia University Institutional Animal Care and Use Committee (Assurance #AAAH8959). Tagged penguins were paired and had brood-stage nests containing two chicks. Penguins were equipped with SPOT 5 satellite transmitters (Wildlife Computers Redmond, WA, USA) and Lotek LAT1400 time-depth recorder (Lotek Wireless, Inc, St. John’s Canada; resolution of 0.05 m, accuracy of 2 m) sampling at 2 Hz. Dive depths less than 5 m were not recorded to save space on the memory cards. Transmitters were attached to the anterior feathers on the lower dorsal region using waterproof tape and small plastic zip ties. Transmitters were removed and rotated to new penguins every 3–5 days dependent on weather conditions. Penguin locations were filtered to remove inaccurate location data due to erroneous terrestrial positions, unreasonable locations based on swimming speed and coastal geometry, following published the data processing methods^37^. We time-matched dive records to location data and the maximum dive depth was determined for each dive. Penguin dives were classified into transit, search and foraging dives, where foraging dives consisted of wiggles, plateaus or bottom time where prey was likely pursued (see^37^ for more information). The convex hulls of penguin locations were computed using chull in the grDevices package^53^.

### Penguin Habitat Selectivity Statistical Tests

We compared the distribution of penguin ARGOS locations to distributions of available RPD values using two-sided Kolmogorov-Smirnov tests (ks.test in the stats package)^53^ to test for habitat selectivity. Penguin selectivity is inferred from the distributional differences between RPD values at penguin ARGOS locations. Several considerations are needed for comparing penguin ARGOS locations to RPD simulations. For this analysis, we used ARGOS classes 1–3 (estimated accuracy is 350–1000 m, 150–350 m and <150 m, respectively), which have errors similar to, or smaller than the RPD grid cells. Penguins periodically haul-out on sea ice or islands during their foraging trips, so we restricted our analysis to ARGOS locations where the wet sensors were triggered and were within the field of computed RPD values. We used two estimates of available habitat for Adélie and gentoo penguins. The first estimate of available RPD habitat is the entire RPD HFR field over Palmer Deep during the times the penguins were foraging, because both Adélie and gentoo penguins are capable of traversing the entire RPD field in a single foraging trip. The second estimate of available RPD habitat was based on simulated Brownian motion of central place foragers (simm.bb in the adehabitatLT R package)^54^, nesting at the Adélie and the gentoo penguin colonies (Fig. 1). We simulated two penguins per day from each colony, which was similar to the tagging effort during the field season. A 10-year analysis of foraging trip duration showed that these penguins take forage trips up to 48 hours, but most are 6–24 hr^22^. Simulated foraging trip duration was limited to 24 hr in one hour time steps, and the simulated penguin speeds were normally distributed around a mean of 4 km hr^−1^, and a maximum of 8 km hr^−1^ to mimic Adélie and gentoo penguin swimming speeds^55^. Brownian motion is an uncorrelated movement that represents random foragers not selecting for environmental features, remembering previous feeding locations, or cuing off of other environmental proxies. We used these simulated penguin locations as a null metric of available RPD values by a non-selecting central place forager originating from the Adélie and gentoo penguin nesting sites.

The possibility of individual effects in tagging studies is a persistent problem reflected in their foraging trips or individual behavior. To deal with the possibility of foraging trip level effects driving the results of the of the Kolmogorov-Smirnov tests, we systematically withheld individual foraging trips and individual penguins from our analysis to test the sensitivity of our results to individuals foraging trips being withheld from the analysis.

## Data Availability

The HFR datasets are archived and accessible through the United States National HF radar archive housed at the National Oceanic and Atmospheric Administration (NOAA) National Data Buoy Center (NDBC): http://hfradar.ndbc.noaa.gov/. Additionally, the post-processed raw and de-tided total vector maps can be accessed via the Rutgers HFR Environmental Research Division Data Access Program (ERRDAPP) Service: http://hfr.marine.rutgers.edu/. The other datasets including the glider and penguin tagged data are available from the corresponding author on reasonable request, as some of these data are still in use for student dissertations.
